# Supplementary material for: Partial connectomes of labeled dopaminergic circuits reveal non-synaptic communication and axonal remodeling after exposure to cocaine
Source: eLife. 2021 Dec 29;10:e71981. doi: 10.7554/eLife.71981 (PMC8716107; doi:10.7554/eLife.71981)
Supplement: Figure 3—source data 1. — The top row (light blue) represents each data point in the x-axis of Figure 3B that counts the number of axons containing greater than the specified number of varicosities/axon. The left column (light yellow) separates each varicosity type. p-values were calculated by dividing the number of times the simulated axon had a specified varicosity type (yellow column) appear more than the number of specified times (blue row) by the total number of Monte Carlo simulations (100,000). [file elife-71981-fig3-data1.pdf]

Varicosities/axon:	>1	>2	>3	>4	>5
Varicosity Type					
I	0.90	0.70	0.46	0.58	0
II	0.53	0.16	0.07	0.001	0
III	0.94	0.02	0.03	0.006	0
IV	0.94	0.03	0.03	0.007	0
